# Quantity of spontaneous touches to body and surface in very preterm and healthy term infants

**DOI:** 10.3389/fpsyg.2024.1459009

**Published:** 2024-12-18

**Authors:** Sophie Stupperich, Anne-Kathrin Dathe, Abigail DiMercurio, John P. Connell, Nicole Baumann, Marianne Jover, Daniela Corbetta, Julia Jaekel, Ursula Felderhoff-Mueser, Britta Huening

**Affiliations:** ^1^Department of Paediatrics I, University Hospital Essen, University of Duisburg-Essen, Essen, Germany; ^2^Centre for Translational Neuro and Behavioural Sciences, c-TNBS, Faculty of Medicine, University Duisburg-Essen, Essen, Germany; ^3^Department of Health and Nursing, Occupational Therapy, Ernst-Abbe-University of Applied Sciences Jena, Jena, Germany; ^4^Department of Psychological Sciences, Kennesaw State University, Kennesaw, GA, United States; ^5^Department of Psychology, University of Tennessee, Knoxville, TN, United States; ^6^Department of Pediatrics, University Hospital Würzburg, Würzburg, Germany; ^7^School of Psychological Sciences, Monash University, Clayton, VIC, Australia; ^8^Department of Population Health Sciences, University of Leicester, Leicester, United Kingdom; ^9^Department of Psychology, University of Warwick, Coventry, United Kingdom; ^10^Centre de Recherche en Psychologie de la Connaissance, du Langage et de l'Émotion, Aix-Marseille Université, Marseille, France; ^11^Faculty of Education and Psychology, Department of Psychology, University of Oulu, Oulu, Finland; ^12^Department of Psychology, University of Copenhagen, Copenhagen, Denmark

**Keywords:** preterm infants, motor development, spontaneous touches, very low birthweight, healthy term infants

## Abstract

**Background:**

Spontaneous movements are a crucial part of early motor development. Healthy term infants may produce up to 200 spontaneous touches to their body and surface in 10 minutes with their hands. The existing literature shows differences in early motor development between very preterm (<32 weeks gestation) and healthy term infants. It is not known whether the quantity of spontaneous touches differs between very preterm infants and healthy term infants.

**Aims:**

This study investigates whether the overall quantity of spontaneous touches to body and surface is lower among low-risk very preterm infants compared with healthy term infants.

**Methods:**

Videos of 25 low-risk very preterm infants (10 female) at a mean corrected age of 13 weeks [Mean = 12.76, Standard Deviation (SD) = 1.07] were recorded during clinical routine and compared with videos of five healthy term infants (chronological mean age = 9.00, SD = 0.63). Spontaneous touches of both hands were coded, assessing number and location of each touch (i.e., body vs. surface).

**Results:**

Very preterm infants showed significantly fewer overall touches per minute (Mean = 8.87, SD = 4.13) than healthy term infants (Mean = 13.19, SD = 4.28), 95% Confidence Interval (CI) = [0.00, 6.84] (*p* = 0.029).

**Discussion and conclusion:**

This study shows that low-risk very preterm infants, on average, produce fewer spontaneous touches than healthy term infants at three months of corrected age. The present study provides important exploratory evidence for further studies, particularly longitudinal investigations of all dimensions of development.

## 1 Introduction

The very first movements of a human embryo can be observed as soon as motor neurons form, which is at around six to nine weeks of gestation (Prechtl, 1985). By moving their extremities and touching the environment surrounding them, embryos and fetuses learn about their own movements, positions and body parts (Fagard et al., 2018). The self-touch behavior infants show from birth is the continuation of what fetuses display *in utero*: a gathering of proprioceptive and haptic information, performed supposedly for gaining sensorimotor experience and learning how and where to guide a touch. Infants learn from different trajectories in which trial and error are crucial (Corbetta and Thelen, 1996). Spontaneous movements could function as a recalibration of movements in the aerial ambient environment as gravitational forces are disruptive to newborns’ movement organization (Fagard et al., 2018). Additionally, infants develop a sense of differentiating themselves from their environment, which is important for distinguishing contacts directed to oneself versus others. At the age of two to three months, most infants have learned to recognize the perception of themselves and their body within their surrounding space (Rochat, 1998). This is called the ‘sense of body’ (Filippetti et al., 2015).

As part of their universal repertoire of behaviors, infants produce many spontaneous movements prior to reaching and grasping (Von Hofsten, 1993; DiMercurio et al., 2018). Spontaneous movements are one of the earliest movements that can be examined and may build the foundations for self-touch behavior and the development of body awareness (Thomas et al., 2014). A part of the spontaneous movement repertoire from nine weeks gestational age to three to five months post term are general movements (GMs), as described by Prechtl (Prechtl, 1997). At the origins of GMs are central pattern generators in the brain, that elicit a variety of spontaneous movements independent of any sensory or motor stimuli. These spontaneous movements evolve and develop over time (Einspieler et al., 2021). At first, central pattern generators cause small side-to-side movements of the head as hiccupping, stretching, suckling or yawning. These small movements progress to more complex spontaneous movements including age-specific GMs and fidgety movements (Bruggink et al., 2009; Einspieler and Prechtl, 2005). Fidgety movements are characterized as small movements with varying acceleration of neck and trunk, often involving the limbs (Einspieler et al., 2016b; Einspieler et al., 2008; Einspieler and Prechtl, 2005; Prechtl et al., 1997). GMs with adequate variability are predictive of a well-functioning, intact nervous system (Einspieler et al., 1997). The absence of fidgety movements around 9–15 weeks post term, for example, are a strong predictor of cerebral palsy (Einspieler et al., 1997; Einspieler et al., 2016a; Einspieler et al., 2004; Einspieler et al., 2016b; Einspieler et al., 2021).

Focusing on self-touch behavior, DiMercurio et al. (2018) studied a sample of five healthy term infants followed weekly from three weeks until two months of age. The authors observed and interpreted spontaneous touches to the body and the environment in different settings (baseline, toys in view). Infants spent about 50% of the time moving their hands from place to place, producing up to 200 spontaneous touches with both hands over a ten-minute interval. In doing so, they contacted all the body areas they could reach to within arm length (i.e., self-touches), including the neighboring surface areas. The duration of those contacts accounted for the remaining 50% of the time. DiMercurio et al. (2018) suggested that healthy term infants were actively exploring their own body during touch as well as the space around them when moving their arms from place-to-place and that these sensorimotor experiences are foundational for developing future motor behaviors.

Altogether, the findings reviewed above mainly apply to healthy term infants from normative population samples. Studies on spontaneous touches of infants born preterm are rare. Data on spontaneous touches during infants’ fidgety movement’s age range could help better understand the neurodevelopmental mechanisms contributing to delayed or impaired motor behavior. Neonatal intensive care has advanced over the past decades. As a result, the limit of viability for infants born preterm is now 22 weeks of gestation (Suarez-Idueta et al., 2023). However, the long-term morbidity of those at highest neurodevelopmental risk, infants born very (28–32 weeks of gestation) and extremely preterm (<28 weeks of gestation) has not decreased to the same extent as mortality (Suarez-Idueta et al., 2023; Marlow et al., 2021; Cheong et al., 2017).

Evidence show that very preterm infants without neurological impairments experience motor difficulties much more often than term born controls in later childhood (Baumann et al., 2020; de Kieviet et al., 2009). Very preterm infants do not perform as well as healthy term infants concerning motor functions, especially in the first two years of life (Einspieler et al., 2016a; Babik et al., 2017; Örtqvist et al., 2021). The current literature suggests that disruptions in very preterm infants’ basic motor abilities may be related to posture issues such as extended positions, retracted shoulders, and atypically extended arms, indicating muscular hypotonia in the shoulders and trunk (Madlinger-Lewis et al., 2014; Dusing et al., 2005; Örtqvist et al., 2021). In typically developing term born infants, the extended arm posture is generally adopted around 2 months of age (DiMercurio et al., 2022). As a result of their early arm extended posture, very preterm infants may have difficulties in holding hands in the midline leading to reduced hand-to-mouth contact as well as limited arm lifting against gravity and reduced visual interaction with their hands compared to healthy term infants (Babik et al., 2017). This may limit touches to the own body. When looking at the extremities, distance and control over movements may be reduced in very preterm infants (Delafield-Butt et al., 2018), which could reduce the range of movements. Possibly associated with difficulties in arm lifting may be a reduced explorative behavior with less touches to the body (Babik et al., 2017; Thomas et al., 2014). In some very preterm infant samples, exploration of the body, posture and midline crossing, fidgety movements, movement patterns and motor repertoire are rated as abnormal or not age-adequate (Örtqvist et al., 2021). In the study performed by Örtqvist et al., very preterm infants showed a higher rate of abnormal early motor performance and in total there was a significant difference between the groups in every sub-category of the revised ‘Motor Optimality Score – Revised’ of GMs (Örtqvist et al., 2021).

In summary, prior observations have clearly shown a delay in early motor development in very preterm infants, however, the mentioned studies until now have only compared healthy term infants to very preterm infants who suffered a variety of severe postnatal complications (e.g., necrotizing enterocolitis, intra-cerebral hemorrhage, intra-ventricular hemorrhage, bronchopulmonary dysplasia) (Heineman et al., 2008). It remains unclear whether it is the very preterm birth itself or the superimposed postnatal complications that are associated with the observed developmental difficulties. No study to date has investigated a sample of very preterm infants without severe neurological and medical complications. However, Madelaine et al. tested children who were born preterm and seemed at low risk of neurological disorders. The authors found that later during school age years, the children displayed different visual-motor patterns compared to same age full term peers (Madelaine, 2019; Madelaine et al., 2021). By studying such population, it is possible to disentangle the effects of disrupted gestation on spontaneous touches from other confounding effects or other superimposed risk factors. Therefore, this study investigates spontaneous touches of low-risk very preterm infants compared to healthy term infants.

Based on the disruption in spontaneous movements and motor delays observed around the age when fidgety movements can be observed, we assessed the overall quantity of spontaneous touches to body and surface in very preterm infants at 13 weeks corrected age compared to healthy term infants aged about 9 weeks. If prematurity alone, without other confounding factors, matters, we hypothesize that low-risk very preterm infants should show a lower quantity of overall spontaneous touches to their body and surface than healthy term infants. We additionally explored whether low-risk very preterm infants directed fewer self-touches to their own body than healthy term infants and fewer touches to their body than the surrounding surface.

## 2 Materials and methods

### 2.1 Participants

**Figure 1:**
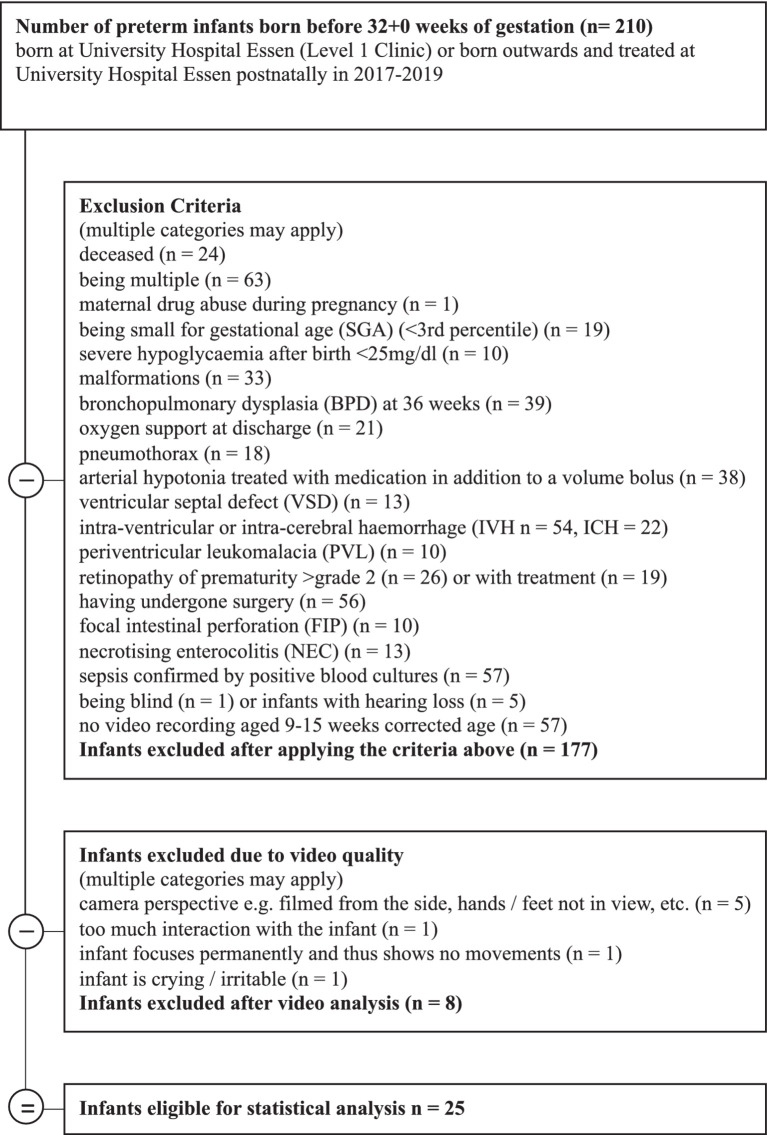
Recruitment strategy and exclusion criteria.

Included in the study were very preterm infants born before 32 + 0 weeks of gestation in the years 2017–2019. The infants were born or treated postnatally at a hospital in an urban region of western Germany and assessed with video recordings used for GM analysis at 9 to 15 weeks corrected age (chronological age in weeks minus weeks born prematurely). Infants with major co-morbidities were excluded, 25 very preterm infants at mean age 12.76 weeks, (SD = 1.07, range = 11–16) fulfilled the inclusion criteria (see Figure 1). Parents of the very preterm infants gave their written informed consent to the recording and analysis of videos for assessing motor development. These videos were analyzed retrospectively with regard to spontaneous touches to their body and the surface they laid on for this study. Ethics approval was obtained from the Ethics Committee of the University of Duisburg-Essen (18-8388-BO). Healthy term infants (gestational age at least 37 + 0 weeks) recruited in the study performed by DiMercurio et al. (2018) (*n* = 4) plus 5th baby collected at later date were used as a control group (DiMercurio et al., 2018). Healthy term infants were 8 to 10 weeks old (chronological age), (Mean = 9.00, SD = 0.63, ranges = 8–10). Videos of the healthy term infants were obtained in the years 2016–2018 in a mid-sized city in the south of the United States (US) (DiMercurio et al., 2018). Parents gave their written informed consent to participate in the study. Ethics approval was obtained from the Institutional Review Board of the University of Tennessee, Knoxville (15-02158-FB).

### 2.2 Materials

Videos of the very preterm infants were recorded using a Sony Handycam Camera (HDR-CX402 and HDR-CX405). For the healthy term infants, videos were recorded using a Panasonic (PV-GS39) camera. For the healthy term infants, multiple longitudinal recordings across different conditions were made, but only the videos in baseline condition (no toys, above angle view) that matched the age and conditions of the very preterm infant were used. All infants were placed supine on an all-white foam surface and baseline was always the first condition recorded (DiMercurio et al., 2018).

### 2.3 Procedure

**Figure 2:**
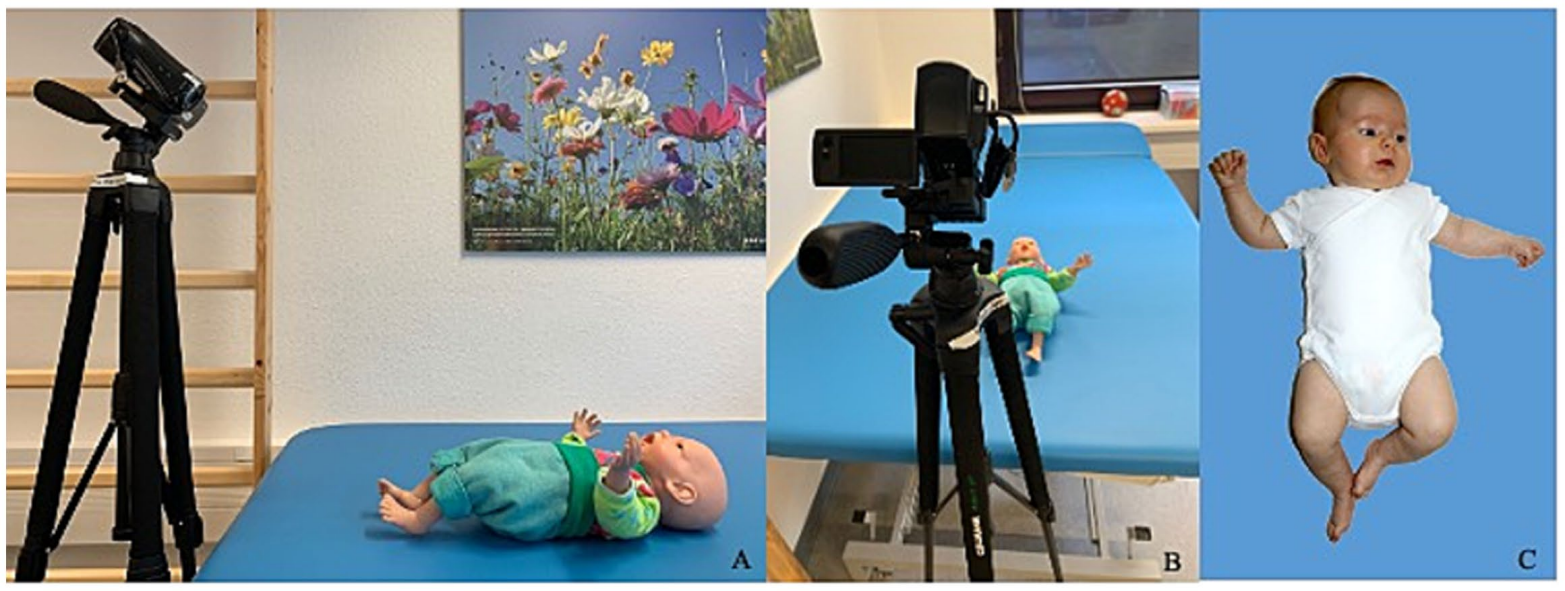
Video recording of the very preterm infant. Positioning of the infant on the surface for video recording. The infant is lying in a supine position, filmed at a 45° angle **(A,B)**. Wrists and ankles need to be visible. When looking at the video the infant was visible as seen in **(C)**.

Video recordings of the very preterm infants were carried out as part of the GM analysis. Thus, in all videos of very preterm infants, there were conversations between parents and medical staff in the same room. As videos for the very preterm infants were obtained during clinical routine, the recording conditions of each video differed slightly (daytime, pattern of underlying surface, clothing of infant, number of people in the room, etc.). For the recordings in both groups, the infant was placed on the examination bed wearing a nappy or onesie. When wearing a onesie or other clothing, long sleeves had to be rolled up, so wrists as well as ankles were visible. The camera was placed on a tripod caudally to the infant, filming at an angle of 45° (see Figure 2). During the recordings, toys were removed. Additionally, interaction with the infant was not recommended if possible. If an infant was irritable, parents were allowed to calm the infant. If possible, the use of a pacifier was avoided, but parents were allowed to give the infant a pacifier when signs of irritability were shown. Sequences of video when infants had a pacifier or parent-infant interaction occurred were not used for analysis. Recordings were three to five minutes long. After removing invalid video sequences, a valid video length for coding of 2 min 30 s had to be reached. All videos were shortened to 2 min 30 s. Videos were coded and analyzed retrospectively.

### 2.4 Video coding of touches and data analyses

Video Coding was performed using the Video Coding Software Datavyu v1.3.7 (Datavyu Team, Databrary Project, New York University).

Videos of both groups were coded using the same software version and following the same protocols. The coding technique was adapted from DiMercurio et al. (2018) but slightly modified to meet the purposes of the present study. Since we were only interested in frequencies, the areas of interest were simplified. Two members of the US team trained one member of the German team in coding the videos. Training videos were not included in the main analyses. To ensure valid coding after completed training, an inter-rater agreement and reliability score was calculated. As in DiMercurio et al. (2018), for each touch an error margin of 280 ms (7 frames) difference per onset and offset was tolerated. For calculating post-training agreement and reliability, four videos (of three minutes length) were used.

At the completion of training, the following agreement was reached: onset agreement for left- and right-hand touches was 98%, offset agreement for left- and right-hand touches was 94%, location agreement for left- and right-hand touches was 94%.

After adequate reliability was established, the analysis of cohort videos was initiated. After all the videos of the very preterm infants (*n* = 25) were coded, five videos from that cohort were randomly chosen to be analyzed again to perform an intra-rater reliability check.

For the healthy term infants (*n* = 5) an inter-rater reliability was performed as videos were allowed to be shared between the Knoxville and Essen team. Cohens Kappa (r) for the very preterm infants was *r* = 0.91 and for the healthy term infants *r* = 0.76.

Sequences of the videos showing interaction (e.g., touch of a parent, pacifier use, etc.) with the infant were not coded. Touches were coded for the right and left hand separately following the same coding scheme as in DiMercurio et al. (2018).

**Figure 3:**
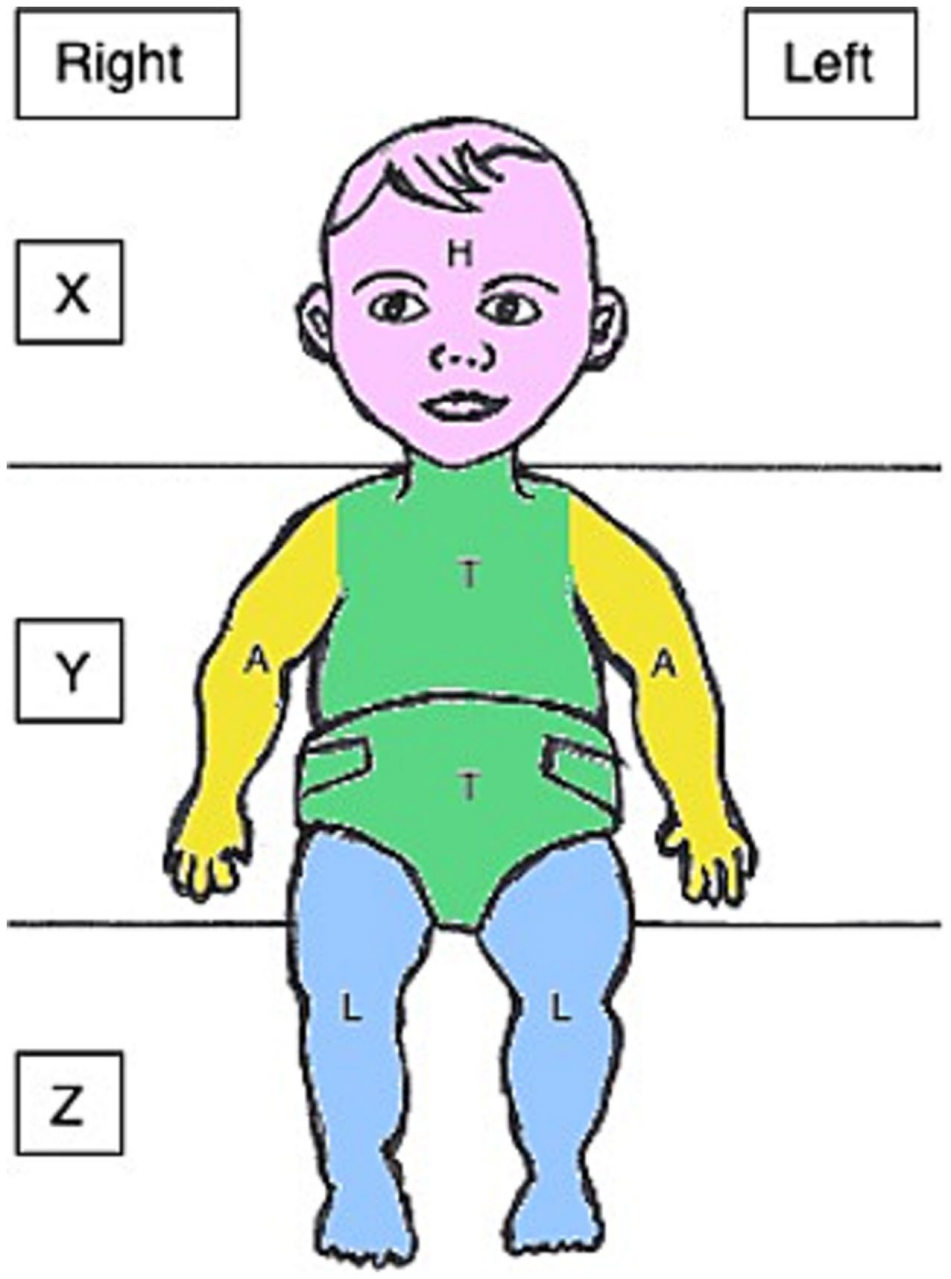
Zones for coding of spontaneous touches on an infant seen from the perspective of the video recording (H, head; T, torso; A, arm; L, legs; X, Y, and Z refer to the surrounding surface areas).

For each touch, the onset, offset and location were marked. Videos were coded frame by frame (40 ms per frame). To be counted as a touch, a contact had to last at least 280 ms (seven frames). By coding on- and offset, the duration of each touch could be evaluated. When marking the location, the surface was divided into three (X, Y, Z) and the body into four different zones (H = head, T = torso, A = arms, L = legs) (see Figure 3). When a touch occurred and the hand moved from one area to another while still maintaining contact to the surface or body a dash (“-“) was used to indicate the area transition within the same touch. Touches where the hand was in between two areas were defined by a slash (“/”). An example of touch transition would be: “H-T-H” with the touch starting at the head, with the hand moving to the torso whilst maintaining contact to the body and then moving back to the head. An example of touch at the border of two location would be: “Y/T” with the hand touching zone Y of the surface and the torso at the same time. A combination of both situations was also possible (e.g.,” H/T–T-Y″). Touches were only coded when the coder was certain that the hand had established contact to a surface or body area. For estimating the overall number of touches between body and surface, the numbers of touches to all body locations (H, T, A, L) and all surface areas (X, Y, Z) were combined.

If the hand could not be seen (e.g., hidden behind a leg) the sequence was marked as an unknown touch (“-999”). Video sequences when it was not clear whether a touch occurred or not were marked as ambiguous touches (“-777”). In most of these situations the hand was placed with the thumb facing upwards. In some cases, wires (due to saturation monitors) or patterns on the sheets placed on the bed made it difficult to clearly differentiate if a hand was touching or not. In the analysis, unknown (−999) and ambiguous (−777) touches were not included. To account for the lost sequences, additional time of the video was included until 02:30 min of coded video time was analyzed for every infant (with valid touches).

### 2.5 Statistical analysis

After completion of the video coding, the data obtained were analyzed using SPSS Version 27.0 (IBM Corp, NY, USA). Mann–Whitney U tests were used to test the hypotheses. Before starting the recruitment process, a power calculation was performed to estimate the sample size needed to obtain acceptable effect sizes. The power analysis for the group comparison was based on the total number of touches corrected for the length of analyzed time. For the right hand the results were as follows: anticipated effect size (Cohen’s d) based on pilot data = 0.83; minimum total sample size (one-tailed hypothesis) = 38; minimum sample size per group (one-tailed hypothesis) = 19. The results for the left hand were similar, as it can be seen as follows: anticipated effect size (Cohen’s d) based on pilot data = 0.89; minimum total sample size (one-tailed hypothesis) = 34; minimum sample size per group (one-tailed hypothesis) = 17. The comparison group of healthy term infants was small but also very homogeneous, so a sample size of 25 very preterm infants was estimated to achieve 80% power.

## 3 Results

Of the very preterm infants included, 15 were female and ten were male (Table 1). Mild co-morbidities of these infants included arterial hypotonia treated with intravenous fluids (*n* = 3), retinopathy of prematurity grade one (*n* = 5), patent ductus arteriosus (PDA) without treatment (*n* = 7), pharmacological treatment of asymptomatic infants during the first week of life (*n* = 5). All PDAs were not classified as being hemodynamically relevant. Only two very preterm infants required mechanical ventilation for 4 and 5 days, respectively. Magnetic resonance imaging (MRI) was performed in all very preterm infants at term equivalent age. This revealed the presence of diffuse excessive high signal intensity (DEHSI) in four infants and isolated punctate lesions in one. Of the healthy term infants, three infants were male, two were female. One infant had an Apgar score of three at one minute and five at five minutes. No co-morbidities were reported. All infants in both groups showed fidgety movements.

**Table 1:** Group characteristics of very preterm and healthy term infants.

	Very preterm infants (*n* = 25)	Healthy term infants (*n* = 5)
Sex [*n* = male/female (%)]	15/10 (60/40)	3/2 (60/40)
Gestational age at birth in weeks [Mean ± SD (range)]	29.64 ± 1.52 (26–31)	37.80 ± 1.10 (37–39)
Birthweight in grams [Mean ± SD (range)]	1374.80 ± 370.39 (770–2050)	3213.00 ± 381.28 (2693–3,742)
Body length at birth in cm [Mean ± SD (range)]	39.08 ± 3.97 (31.00–44.00)	49.71 ± 1.72 (46.99–51.50)
Apgar 1′ [Mean ± SD (range)]	7.04 ± 1.27 (4–9)	6.60 ± 2.19 (3–8)
Apgar 5′ [Mean ± SD (range)]	8.04 ± 0.89 (6–9)	8.20 ± 1.79 (5–9)
Ventilation days [Mean ± SD (range)]	0.36 ± 1.25 (0–5)	0.00 ± 0.00 (0–0)
Age in weeks at recording [Mean ± SD (range)]	12.76 ± 1.07 (11–16)	9.00 ± 0.63 (8–10)

We had hypothesized that very preterm infants would show a lower quantity of overall touches to body and surface compared to healthy term infants. The mean number of touches to body and surface per minute of total time coded (Table 2) was lower for very preterm infants (Mean = 8.87, SD = 4.13) compared with healthy term infants (Mean = 13.19, SD = 4.28) [Mann–Whitney U-test: *U* = 97.00, *p* = 0.029, 95% CI = (0.00, 6.84)], confirming our main hypothesis.

**Table 2:** Quantity of spontaneous touches to body and surface in very preterm and healthy term infants.

		Very preterm infants (*n* = 25)	Healthy term infants (*n* = 5)	Group differences *p*-value [95% CI]
Overall number of touches	per minute of total time coded [Mean ± SD (ranges)]	8.87 ± 4.13 (3.05–15.81)	13.19 ± 4.28 (8.91–19.29)	*p* = 0.029 [0.00, 6.84]
Number of touches to body locations	per minute of total time coded [Mean ± SD (ranges)]	5.65 ± 6.07 (0.00–20.00)	6.69 ± 6.73 (0.00–15.12)	*p* = 0.978 [−8.64, 4.87]

We also explored differences between very preterm and healthy term infants regarding the quantity of spontaneous touches to their own body. The analysis showed no statistically significant difference between very preterm and healthy term infants [*U* = 63.00, *p* = 0.978, 95% CI = (−8.64, 4.87)] (Table 2).

As part of exploratory subsequent analyses, we looked at the distribution of touches within the group of very preterm infants. The average number of spontaneous touches to the surface was higher than touches to the body [*U* = 209.50, *p* = 0.046, 95% CI = (0.02, 5.93)].

## 4 Discussion

This study investigated the overall quantity of spontaneous touches to the body and surface in low-risk very preterm infants compared with healthy term infants. Results showed that, overall, very preterm infants displayed fewer spontaneous touches than healthy term infants. Further, a trend could be observed indicating less spontaneous touches to the body in very preterm compared to healthy term infants. Finally, when focusing on very preterm infants, the mean number of spontaneous touches to the surface was higher than the mean number of spontaneous touches to the body.

These results need to be discussed against the background of previous studies regarding posture and movement patterns of preterm infants: extended body posture (Örtqvist et al., 2021; Dusing et al., 2005), reduced lifting of arms against gravity (Babik et al., 2017), predominant extension of arms (Örtqvist et al., 2021) and difficulty holding hands at the midline (Babik et al., 2017). The infants included here were born very preterm, but otherwise suffered no severe complications (low-risk). The reduced overall quantity of spontaneous touches in very preterm infants in the present study could be associated with both posture and movement (De Groot, 2000). Possible associated factors for the difference in overall quantity of spontaneous touches in very preterm and healthy term infants could be a predominantly extended posture with predominantly extended arms, the weeks of pregnancy missed and thus the lack of learning crucial *in-utero* skills as well as the first weeks of life and neonatal intensive care unit (NICU) care or altered brain development.

The extended positioning of arms was also observed in this study, with very preterm infants predominantly touching the surface. In the case of muscular hypotonia and reduced trunk control, a lack of arm and trunk flexion and fewer number of touches directed toward the body may help to increase stability. Accordingly, a longitudinal study by Pin et al. (2020) described a delay in segmental trunk control in very preterm infants at four to twelve months compared to a control group. A study performed by Delafield-Butt et al. (2018) looking at arm movements in healthy term and very preterm infants came to the same conclusion. Very preterm infants showed reduced control of arm movements (Delafield-Butt et al., 2018). A lack of organization of touch may result in short touches close to the point of the resting arm. In this current study, very short sequences below 280 ms were not counted as a touch and thus do not explain a reduced number in overall quantity of overall touches. In contrast to what was seen in the very preterm infants in this study, the same healthy term infants observed by DiMercurio et al. (2022) initially kept their arms with a flexed elbow. Thus, healthy term infants had a diverse movement repertoire with hand to head orientation and gradually moved on to a predominant extended arm posture with hand to feet orientation only at around 2 months of age.

One approach to explaining the difference in overall quantity of spontaneous touches to body and surface shown in this study may be looking at discrepancies at the fetal stage. A study by Marx and Nagy (2015) showed a difference in the development of fetal movements according to gestational age. In the third trimester fetuses showed more spontaneous movements than in the second trimester. Furthermore, as space *in utero* became more and more limited as the pregnancy proceeded, in the third trimester more movements to the body, especially face and mouth, were observed than in the second trimester. Movements with crossing arms saved space and were observed more commonly in fetuses in the third than in the second trimester (Marx and Nagy, 2015; Fagard et al., 2018). The third trimester is a period of pregnancy which all of the very preterm infants included in this study lacked. As very preterm infants were born at a mean gestational age of 29.64 weeks, they did not experience the phase of pregnancy where space is limited and self-touch becomes inevitable (Piontelli, 1987). Ex-utero very preterm infants lack the surrounding space which *in utero* restricts and thus guides their movements toward their own body and midline. In the present study very preterm infants showed less spontaneous touches than healthy term infants. Thus, it can be speculated, that very preterm infants lack the specific motor developmental skills achieved through environmental conditions in later pregnancy. This should be further explored in future studies to open potential avenues toward intervention during the first few weeks after a very preterm birth.

A different approach to explaining the difference in overall quantity of spontaneous touches to body and surface could be an alteration or delay of motor skills developed due to NICU care. There is considerable difference in how very preterm and healthy term infants spend their first weeks and months of life. While healthy term infants usually go home with their parents within a few days after birth and can focus on adapting and developing, very preterm infants are kept in observation and under care at the intensive neonatology hospital unit for a much more extended time and struggle with far more crucial elements of life such as breathing, feeding, and regulating their metabolic functions. Many very preterm infants need respiratory support, parenteral feeding, injections, and other interventions. Although in the present study major organic complications have been excluded, all of the included very preterm infants needed intensive neonatal care. In the NICU, very preterm infants spend most of their time in a supine position. It is a well-known intervention to adapt positioning of the very preterm infants to improve neurodevelopmental, motor, and feeding outcomes. It is important to support a flexed position, as it mimics the position *in utero* in the third trimester and includes flexion of the major joints. A flexed position supports body symmetry and adequate alignment of joints (Madlinger-Lewis et al., 2014; Aucott et al., 2002). Apart from the method of positioning used, very preterm infants receiving neonatal care spend much more time in an extended position, especially if they have trouble breathing, than infants at home who get picked up, carried and nursed via breast- or bottle-feeding.

Another factor influencing motor development in very preterm infants could be maternal care, especially physical contact. It is known that preterm infants react to kangarooing and skin-to-skin contact by optimizing their heart rate and oxygen saturation (Conde-Agudelo et al., 2014; Campbell-Yeo et al., 2015). There are initial indications that the primary motor cortex is more stimulated in children with skin-to-skin contact than in children without (Schneider et al., 2012). An improvement in motor skills through intensive maternal care would be conceivable, but was not part of this study. It is assumed that in preterm infants, the altered experiences with their own body and the environment in the first weeks of life (Peng et al., 2009; Durier et al., 2015) could influence the later body schema, body image and basic sense of body compared to term born infants (Butti et al., 2020; DiMercurio et al., 2018). Visual and proprioceptive information as well as multimodal sensorimotor integration could play a role in this regard (Berlucchi and Aglioti, 2010). One way to enhance sensori motor development in very preterm infants may be by using infant massage. Especially when performed by parents it may positively influence sensorimotor development as a study by Lu et al. (2018) examining infants with motor developmental delay has shown. Parental affectionate touch has shown to lead to a decrease in heart rate in preterm infants by enhancing parasympathetic regulation (Puschel et al., 2022). Although the more preterm the infant the more immature the C-tactile afferent nerve fibers, by which the decrease in heart rate is caused seems to be. Leading to the conclusion that very preterm infants benefit more from affectionate touch than extremely pretem infants (Puschel et al., 2022). Apart from a reduction in heart rate parental touch may have positive effects on somatosensory development and also on autonomic regulation (Carozza and Leong, 2021). Using parents as emotional co-regulators was already defined by Bion with the concept of containment in 1962 by Bion (1962). NICU environment as well as the circumstances of preterm birth may also cause alterations in brain development. Even when looking at a low-risk cohort, impaired brain development could also be associated with the differences between healthy term infants and very preterm infants described in this study. Brain development differs between healthy very preterm infants and healthy term infants. Within the first two years of life the development of the brain is a dynamic and complex process, which is easily influenced by different factors (Knickmeyer et al., 2008). Total brain volume for example increases in 101% within the first year of life and cerebellar volume more than doubles (Knickmeyer et al., 2008). Even in the absence of severe neurological complications (e.g., periventricular leukomalacia, intraventricular hemorrhage, intracerebral hemorrhage) very preterm infants show two main alterations in brain development at term equivalent age: Small biparietal width (in 31% of very preterm infants) and increased interhemispheric distance (in 34% of very preterm infants) (Huning et al., 2018). Reduced biparietal width, including a reduction in deep grey and cortical grey matter, and increased interhemispheric distance indicate impaired brain growth and disproportionate brain to skull growth, respectively (Huning et al., 2018). These findings were associated with lower gestational age and necessary interventions of NICU care as dexamethasone use, prolonged parenteral nutrition, or oxygen support at 36 weeks (Kidokoro et al., 2014). Three different parts of brain development may be especially vulnerable to environmental influences in the NICU, neuronal organization and elaboration of dendrites, glial cell proliferation and maturation, and myelination of corticospinal tracts (Volpe, 2009). Accordingly, Butti et al. (2020) showed differences in very preterm and healthy term infants at the age of six to eight years regarding the perception of their own body and body schema. The authors presumed the underlying cause of the malfunction in former very preterm infants to be an impaired neuronal development.

### 4.1 Strengths, limitations and perspectives

The present study has many strengths such as the strict inclusion criteria and the extensive video analysis. The detailed selection of valid video sequences and thorough coding are especially a strength of the study. However, we must elaborate on some limitations of this study regarding sample size, recording setup and differences *in camera* angles. The sample size between the two groups (healthy term infants *n* = 5; very preterm infants *n* = 25), differs by 20 infants. A power calculation was performed before analysis to minimize bias. Videos of the very preterm infants were recorded during clinical routine and thus followed the standard recording procedure used for the assessment of GM analysis [for references regarding GM analysis video recording see Einspieler et al. (1997) and Einspieler et al. (2016a)]. Videos of the healthy term infants in contrast were recorded in a laboratory setting, designed for the study of DiMercurio et al. (2018). Thus, the video set up and recordings differed slightly, which may have affected data analysis. For instance, the clothing of the infants, wrist sensors in the healthy term infants and camera angles might all have been factors creating unevenness between recordings. In a study by Durier et al. (2015) infants showed more touches to their own body and the environment when wearing light clothing presumably because less clothing enhanced self-soothing behavior (Durier et al., 2015). Following Durier et al.’s findings, the differences in settings between groups in the present study could have been beneficial for the very preterm infants allowing for more touches compared to a setting where very preterm infants would have been fully clothed (as in the healthy term infants). But still, with less clothing, very preterm infants showed less touches than the healthy term infants. Thus, the impact of clothing differences between groups may be negligible. The same can be said about the impact of wrist sensors worn by the healthy term infants during recording (DiMercurio et al., 2018). Parent interaction was reduced to a minimum in both settings. This is important as recent studies indicate that parental touch reduces heart rate of the infant, increases oxygen saturation and causes relaxation, which may contribute to reduce movements (Puschel et al., 2022).

In order to investigate a possible influence of very preterm birth on spontaneous touches, a group of healthy term infants was used as a control. To be precise, very preterm and term infants were examined. It is known that early term infants born before 39 + 0 weeks of gestational age can be affected by respiratory morbidities (Bulut and Buyukkayhan, 2021; Committee on Obstetric Practice, 2013). However, no infant in the healthy term group needed to be ventilated. The mean age of very preterm and healthy term infants at the point of recording differed slightly, however, this is probably negligeable. DiMercurio and colleagues reported that the number of spontaneous touches to the body and the surface did not show a statistically significant developmental trend over the 3 to 12 week period (DiMercurio et al., 2018).

The difference *in camera* angles is a more relevant limitation of this study. The healthy term infants were filmed using an ‘above view’ and a ‘side view’ (DiMercurio et al., 2018), while the very preterm infants were filmed from a single view only. Whilst very preterm infants were filmed at 45°, the recording angle used when filming the healthy term infants was steeper, which made it more difficult for coders to identify a movement as a touch. This was especially the case with touches of the lateral part of the hand. To avoid bias only the above angle view was used for the analyses in this study.

The present study demonstrates differences in the overall quantity of spontaneous touches to the body and surface - a crucial behavior in early motor development - between low-risk very preterm infants and healthy term infants. Although there is not much literature on the long-term effects of motor difficulties in low-risk very preterm infants, studies focusing on the consequences of impairments in early motor development describe reduced motor skills in children at primary and preschool age (Allotey et al., 2018; Baumann et al., 2020; Butti et al., 2020; Madelaine, 2019). According to Dathe et al. (2020), very preterm infants at school age compared to their healthy term born peers had statistically significant greater difficulties in tasks requiring fine motor and visual-motor skills reducing daily functioning and predisposing them to difficulties at school. Baumann et al. (2020) found that very preterm infants showed reduced motor skills still at preschool and primary school age. Later in life, adults who had been born very preterm displayed poorer motor competence or were unable to perform certain movements. This was not only the case in children or adults who suffered neurological complications but also in those with no neurological impairments (Baumann et al., 2020).

In a low-risk cohort of very preterm infants, the hypothesis was confirmed that prematurity alone leads to a lower overall quantity of spontaneous touches to the body and surface compared to healthy term infants. The second assumption, that very preterm infants would show a lower quantity of self-touches, was also confirmed.

It is too early to conclude that the differences in spontaneous touches observed in the present study are an initial sign of delay in early motor developmental in very preterm infants. However, they may be an indicator of developmental difficulties in subsequent motor milestones. The immediate ones to follow spontaneous touch are reaching and grasping skills. If very preterm infants affected by delays in spontaneous touch behavior were to develop problems in reaching and grasping, it may not only affect the development of motor skills but further cascade into cognitive skills. Early motor skills as reaching, grasping, and manual exploration were found to be important predictive markers of cognitive development, as they influence hand-to-eye coordination, attention, memory, and language skills [see for example, Corbetta and Dimercurio (n.d.)]. In the long-term, children who have been very active and showed high level of motor skills in early infancy tend to have better motor skills in later childhood as well as better cognitive skills. This may positively influence academic as well as social skills (Dathe et al., 2020, Adolph and Hoch, 2019, Libertus and Violi, 2016, Needham and Libertus, 2011).

Future directions should aim to substantiate the present findings with larger cohorts. Another important aim would be to follow longitudinally how spontaneous touches in very preterm infants transition into other behaviors all the way through childhood. As for clinical implications that could follow more conclusive research evidence, practitioners should consider implementing therapeutic regimes that positively influence motor development in very preterm infants, independent of neurological comorbidities. Some speculative examples include promoting a flexed position, supporting muscular strength in shoulders and trunk, and involving parents in the care of the infant supporting self-touch.

This study shows that low-risk very preterm infants, on average, produce fewer spontaneous touches to their bodies and proximal surface than healthy term infants. This can already be observed at a corrected age of three months. The present study provides important explorative pointers for further studies, particularly longitudinal investigations of all dimensions of development.

## Data Availability

The datasets presented in this article are not readily available because data contains videos of infants in which they can be identified as their faces are visible. Sharing these videos with individuals outside the study group was not permitted by parents. Requests to access the datasets should be directed to Sophie Stupperich, sophie@stupperich.eu.

## References

[ref1] Adolph K. E. Hoch J. E. (2019). Motor development: embodied, embedded, enculturated, and enabling. Annu. Rev. Psychol. 70, 141–164. doi: 10.1146/annurev-psych-010418-102836, PMID: 30256718 PMC6320716

[ref3] Allotey J. Zamora J. Cheong-See F. Kalidindi M. Arroyo-Manzano D. Asztalos E.. (2018). Cognitive, motor, behavioural and academic performances of children born preterm: a meta-analysis and systematic review involving 64 061 children. BJOG 125, 16–25. doi: 10.1111/1471-0528.14832, PMID: 29024294

[ref4] Aucott S. Donohue P. K. Atkins E. Allen M. C. (2002). Neurodevelopmental care in the Nicu. Ment. Retard. Dev. Disabil. Res. Rev. 8, 298–308. doi: 10.1002/mrdd.10040 12454906

[ref5] Babik I. Galloway J. C. Lobo M. A. (2017). Infants born preterm demonstrate impaired exploration of their bodies and surfaces throughout the first 2 years of life. Phys. Ther. 97, 915–925. doi: 10.1093/ptj/pzx064, PMID: 28605484

[ref6] Baumann N. Tresilian J. Bartmann P. Wolke D. (2020). Early motor trajectories predict motor but not cognitive function in preterm- and term-born adults without pre-existing neurological conditions. Int. J. Environ. Res. Public Health 17:3258. doi: 10.3390/ijerph17093258, PMID: 32392779 PMC7246453

[ref7] Berlucchi G. Aglioti S. M. (2010). The body in the brain revisited. Exp. Brain Res. 200, 25–35. doi: 10.1007/s00221-009-1970-7 19690846

[ref8] Bion W. (1962). Learrning from experience. London: Heinemann.

[ref9] Bruggink J. L. Cioni G. Einspieler C. Maathuis C. G. Pascale R. Bos A. F. (2009). Early motor repertoire is related to level of self-mobility in children with cerebral palsy at school age. Dev. Med. Child Neurol. 51, 878–885. doi: 10.1111/j.1469-8749.2009.03294.x, PMID: 19416326

[ref10] Bulut O. Buyukkayhan D. (2021). Early term delivery is associated with increased neonatal respiratory morbidity. Pediatr. Int. 63, 60–64. doi: 10.1111/ped.14437 32786118

[ref11] Butti N. Montirosso R. Giusti L. Borgatti R. Urgesi C. (2020). Premature birth affects visual body representation and body schema in preterm children. Brain Cogn. 145:105612. doi: 10.1016/j.bandc.2020.105612, PMID: 32890903

[ref12] Campbell-Yeo M. L. Disher T. C. Benoit B. L. Johnston C. C. (2015). Understanding kangaroo care and its benefits to preterm infants. Pediatr. Health Med. Ther. 6, 15–32. doi: 10.2147/PHMT.S51869 PMC5683265 29388613

[ref13] Carozza S. Leong V. (2021). The role of affectionate caregiver touch in early neurodevelopment and parent–infant interactional synchrony. Front. Neurosci. 14:613378. doi: 10.3389/fnins.2020.613378, PMID: 33584178 PMC7873991

[ref14] Cheong J. L. Y. Anderson P. J. Burnett A. C. Roberts G. Davis N. Hickey L.. (2017). Changing neurodevelopment at 8 years in children born extremely preterm since the 1990s. Pediatrics 139. doi: 10.1542/peds.2016-4086 28814550

[ref15] Committee on Obstetric Practice (2013). ACOG Committee opinion no 579: definition of term pregnancy. Obstet. Gynaecol. 122, 1139–1140. doi: 10.1097/01.AOG.0000437385.88715.4a 24150030

[ref16] Conde-Agudelo A. Jm B. Diaz-Rossello J. (2014). Kangaroo mother care to reduce morbidity and mortality in low birthweight infants. Cochrane Database Syst. Rev. 3:11. doi: 10.1002/14651858.CD002771.pub3 24752403

[ref17] Corbetta D. Dimercurio A. (n.d.). “Manual exploration and the acquisition of skills” in Handbook of perceptual development. ed. Johnson S. (New York: Oxford University Press).

[ref18] Corbetta D. Thelen E. (1996). The developmental origins of bimanual coordination: a dynamic perspective. J. Exp. Psychol. Hum. Percept. Perform. 22, 502–522. doi: 10.1037/0096-1523.22.2.502, PMID: 8934856

[ref9002] Dathe A. K. Jaekel J. Franzel J. Hoehn T. Felderhoff-Mueser U. Huening B. M. (2020). Visual perception, fine motor, and visual-motor skills in very preterm and term-born children before school entry–observational cohort study. Children 7:276. doi: 10.3390/children712027 33291494 PMC7762188

[ref19] De Groot L. (2000). Posture and motility in preterm infants. Dev. Med. Child Neurol. 42, 65–68, PMID: 10665978 10.1017/s0012162200000128

[ref20] De Kieviet J. F. Piek J. P. Aarnoudse-Moens C. S. Oosterlaan J. (2009). Motor development in very preterm and very low-birth-weight children from birth to adolescence: a meta-analysis. JAMA 302, 2235–2242. doi: 10.1001/jama.2009.1708 19934425

[ref21] Delafield-Butt J. T. Freer Y. Perkins J. Skulina D. Schögler B. Lee D. N. (2018). Prospective organization of neonatal arm movements: a motor foundation of embodied agency, disrupted in premature birth. Dev. Sci. 21:e12693. doi: 10.1111/desc.12693, PMID: 29920860 PMC6220947

[ref22] DiMercurio A. Connell J. P. Clark M. Corbetta D. (2018). A naturalistic observation of spontaneous touches to the body and environment in the first 2 months of life. Front. Psychol. 9:2613. doi: 10.3389/fpsyg.2018.02613, PMID: 30619012 PMC6305473

[ref23] Dimercurio A. Springer C. M. Corbetta D. (2022). “Postures of the arms in the first two postnatal months” in Proceedings of the 2022 IEEE international conference on development and learning (ICDL) (London, United Kingdom), 142–147.

[ref24] Durier V. Henry S. Martin E. Dollion N. Hausberger M. Sizun J. (2015). Unexpected behavioural consequences of preterm newborns' clothing. Sci. Rep. 5:9177. doi: 10.1038/srep09177, PMID: 25776252 PMC4361844

[ref25] Dusing S. Mercer V. Yu B. Reilly M. Thorpe D. (2005). Trunk position in supine of infants born preterm and at term: an assessment using a computerized pressure mat. Pediatr. Phys. Ther. 17, 2–10. doi: 10.1097/01.PEP.0000154106.52134.80, PMID: 16357652

[ref26] Einspieler C. Bos A. F. Libertus M. E. Marschik P. B. (2016a). The general movement assessment helps us to identify preterm infants at risk for cognitive dysfunction. Front. Psychol. 7:406. doi: 10.3389/fpsyg.2016.00406 27047429 PMC4801883

[ref27] Einspieler C. Pb M. Hfr P. (2008). Human motor behavior. Prenatal origin and early postnatal development. J. Psychol. 216, 147–153. doi: 10.1027/0044-3409.216.3.147

[ref28] Einspieler C. Peharz R. Marschik P. B. (2016b). Fidgety movements - tiny in appearance, but huge in impact. J. Pediatr. 92, S64–S70. doi: 10.1016/j.jped.2015.12.003, PMID: 26997356

[ref29] Einspieler C. Prechtl H. F. (2005). Prechtl's assessment of general movements: a diagnostic tool for the functional assessment of the young nervous system. Ment. Retard. Dev. Disabil. Res. Rev. 11, 61–67. doi: 10.1002/mrdd.20051, PMID: 15856440

[ref31] Einspieler C. Prechtl H. F. Ferrari F. Cioni G. Bos A. F. (1997). The qualitative assessment of general movements in preterm, term and young infants — review of the methodology. Early Hum. Dev. 50, 47–60. doi: 10.1016/S0378-3782(97)00092-3, PMID: 9467693

[ref30] Einspieler C. Prechtl H. F. R. Bos A. F. Ferrari F. Cioni G. (2004). Prechtl's method on the qualitative assessment of general movements in preterm, Term and Young Infants. London, UK: MacKeith Press. 10.1016/s0378-3782(97)00092-3 9467693

[ref32] Einspieler C. Zhang D. Marschick P. (2021). The significance of fetal and neonatal spontaneous movements for development and early identification of developmental disorders. Kindheit und Entwicklung 30, 6–14. doi: 10.1026/0942-5403/a000323

[ref33] Fagard J. Esseily R. Jacquey L. O'regan K. Somogyi E. (2018). Fetal origin of sensorimotor behavior. Front. Neurorobot. 12:23. doi: 10.3389/fnbot.2018.00023, PMID: 29875649 PMC5974044

[ref34] Filippetti M. L. Orioli G. Johnson M. H. Farroni T. (2015). Newborn body perception: sensitivity to spatial congruency. Infancy 20, 455–465. doi: 10.1111/infa.12083, PMID: 26709351 PMC4682457

[ref35] Heineman K. R. Bos A. F. Hadders-Algra M. (2008). The infant motor profile: a standardized and qualitative method to assess motor behaviour in infancy. Dev. Med. Child Neurol. 50, 275–282. doi: 10.1111/j.1469-8749.2008.02035.x, PMID: 18279412

[ref37] Huning B. Storbeck T. Bruns N. Dransfeld F. Hobrecht J. Karpienski J.. (2018). Relationship between brain function (aeeg) and brain structure (Mri) and their predictive value for neurodevelopmental outcome of preterm infants. Eur. J. Pediatr. 177, 1181–1189. doi: 10.1007/s00431-018-3166-2, PMID: 29789947 PMC6061051

[ref38] Kidokoro H. Anderson P. J. Doyle L. W. Woodward L. J. Neil J. J. Inder T. E. (2014). Brain injury and altered brain growth in preterm infants: predictors and prognosis. Pediatrics 134, e444–e453. doi: 10.1542/peds.2013-2336, PMID: 25070300

[ref39] Knickmeyer R. C. Gouttard S. Kang C. Evans D. Wilber K. Smith J. K.. (2008). A structural Mri study of human brain development from birth to 2 years. J. Neurosci. 28, 12176–12182. doi: 10.1523/JNEUROSCI.3479-08.2008, PMID: 19020011 PMC2884385

[ref40] Libertus K. Violi D. A. (2016). Sit to talk: relation between motor skills and language development in infancy. Front. Psychol. 7:475. doi: 10.3389/fpsyg.2016.00475 27065934 PMC4815289

[ref41] Lu W. P. Tsai W. H. Lin L. Y. Hong R. B. Hwang Y. S. (2018). The beneficial effects of massage on motor development and sensory processing in young children with developmental delay: a randomized control trial study. Dev. Neurorehabil. 22, 487–495. doi: 10.1080/17518423.2018.1537317 30376388

[ref42] Madelaine C. (2019). Effets à long terme de la prématurité sur les habiletés perceptivo-motrices chez des enfants âgés de 8 ans. Psychologie. Français: Normandie Université.

[ref43] Madelaine C. Benguigui N. Molina M. (2021). What do we know about motor development of preterm children without major neurological damage and disorder? A narrative review. J. Motor Learn. Dev. 9, 533–558. doi: 10.1123/jmld.2020-0059

[ref44] Madlinger-Lewis L. Reynolds L. Zarem C. Crapnell T. Inder T. Pineda R. (2014). The effects of alternative positioning on preterm infants in the neonatal intensive care unit: a randomized clinical trial. Res. Dev. Disabil. 35, 490–497. doi: 10.1016/j.ridd.2013.11.019, PMID: 24374602 PMC3938096

[ref45] Marlow N. Ni Y. Lancaster R. Suonpera E. Bernardi M. Fahy A.. (2021). No change in neurodevelopment at 11 years after extremely preterm birth. Arch. Dis. Child Fetal Neonatal Ed. 106, 418–424. doi: 10.1136/archdischild-2020-320650, PMID: 33504573

[ref46] Marx V. Nagy E. (2015). Fetal Behavioural responses to maternal voice and touch. PLoS One 10:e0129118. doi: 10.1371/journal.pone.0129118, PMID: 26053388 PMC4460088

[ref47] Needham A. Libertus K. (2011). Embodiment in early development. Wiley Interdiscip. Rev. Cogn. Sci. 2, 117–123. doi: 10.1002/wcs.109, PMID: 26301917

[ref48] Örtqvist M. Einspieler C. Marschik P. B. Aden U. (2021). Movements and posture in infants born extremely preterm in comparison to term-born controls. Early Hum. Dev. 154:105304. doi: 10.1016/j.earlhumdev.2020.105304, PMID: 33556691

[ref49] Peng N.-H. Bachman J. Jenkins R. Chen C.-H. Chang Y.-C. Chang Y.-S.. (2009). Relationships between environmental stressors and stress biobehavioral responses of preterm infants in Nicu. J. Perinat. Neonatal Nurs. 23, 363–371. doi: 10.1097/JPN.0b013e3181bdd3fd, PMID: 19915421

[ref9001] Pin T. W. Butler P. B. Cheung H. M. Shum S. L. F. (2020). Longitudinal development of segmental trunk control in full term and preterm infants-a pilot study: part II. Dev. Neurorehabilit. 23, 193–200. doi: 10.1080/17518423.2019.1629661 31208258

[ref50] Piontelli A. (1987). Infant observation from before birth. Int. J. Psychoanal. 68, 453–463. 3325439

[ref51] Prechtl H. F. (1985). Ultrasound studies of human fetal behaviour. Early Hum. Dev. 12, 91–98. doi: 10.1016/0378-3782(85)90173-2 3905352

[ref52] Prechtl H. F. (1997). State of the art of a new functional assessment of the young nervous system. An early predictor of cerebral palsy. Early Hum. Dev. 50, 1–11. doi: 10.1016/S0378-3782(97)00088-1, PMID: 9467689

[ref53] Prechtl H. F. Einspieler C. Cioni G. Bos A. F. Ferrari F. Sontheimer D. (1997). An early marker for neurological deficits after perinatal brain lesions. Lancet 349, 1361–1363. doi: 10.1016/S0140-6736(96)10182-3 9149699

[ref54] Puschel I. Reichert J. Friedrich Y. Bergander J. Weidner K. Croy I. (2022). Gentle as a mother's touch: C-tactile touch promotes autonomic regulation in preterm infants. Physiol. Behav. 257:113991. doi: 10.1016/j.physbeh.2022.113991 36242858

[ref55] Rochat P. (1998). Self-perception and action in infancy. Exp. Brain Res. 123, 102–109. doi: 10.1007/s002210050550 9835398

[ref56] Schneider C. Charpak N. Jg R.-P. Tessier R. (2012). Cerebral motor function in very premature-at-birth adolescents: a brain stimulation exploration of kangaroo mother care effects. Acta Paediatr. 101, 1045–1053. doi: 10.1111/j.1651-2227.2012.02770.x 22734793

[ref57] Suarez-Idueta L. Blencowe H. Okwaraji Y. B. Yargawa J. Bradley E. Gordon A.. (2023). Neonatal mortality risk for vulnerable newborn types in 15 countries using 125.5 million nationwide birth outcome records, 2000-2020. BJOG. doi: 10.1111/1471-0528.17506 PMC12678064 37156244

[ref58] Thomas B. L. Karl J. M. Whishaw I. Q. (2014). Independent development of the reach and the grasp in spontaneous self-touching by human infants in the first 6 months. Front. Psychol. 5:1526. doi: 10.3389/fpsyg.2014.01526 25620939 PMC4288059

[ref59] Volpe J. J. (2009). The encephalopathy of prematurity—brain injury and impaired brain development inextricably intertwined. Semin. Pediatr. Neurol. 16, 167–178. doi: 10.1016/j.spen.2009.09.005, PMID: 19945651 PMC2799246

[ref60] Von Hofsten C. (1993). Prospective control: a basic aspect of action development. Hum. Dev. 36, 280–292.

