# Supplementary material for: Quantity of spontaneous touches to body and surface in very preterm and healthy term infants
Source: Front Psychol. 2024 Dec 18;15:1459009. doi: 10.3389/fpsyg.2024.1459009 (PMC11689659; doi:10.3389/fpsyg.2024.1459009)
Supplement: Supplementary file 1 [file Table_1.docx]

Supplementary Material

# Coding scheme

Based on the Coding Scheme by DiMercurio et al. 02/2018


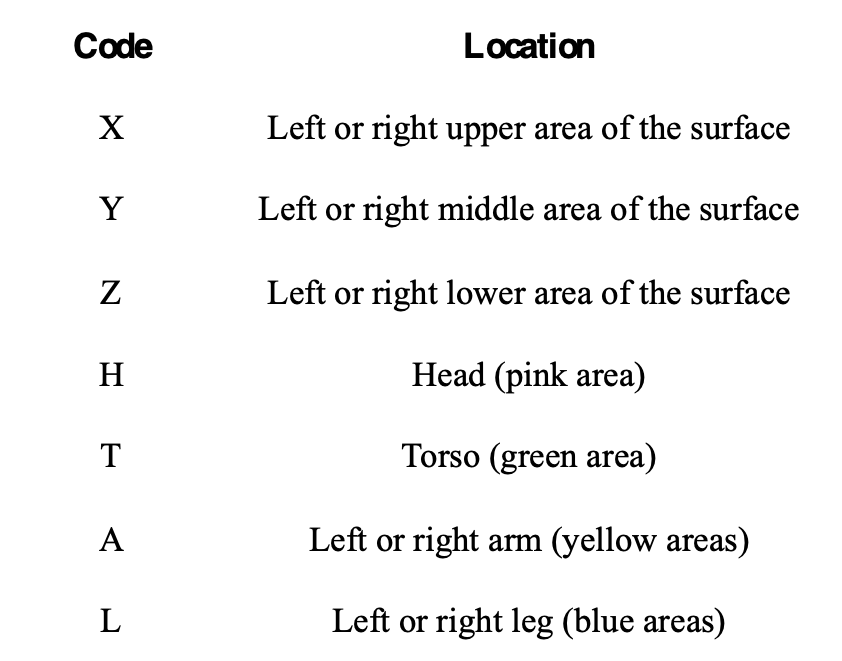


## How to code

- The baby outline above demonstrates the different body areas (targets) a touch of the hands may be aimed at
- Coded are all touches of the left hand and of the right hand individually
- For each touch the following features are coded:
  - Starting point (left or right hand) including timely onset of the touch
  - End point (target of the touch (head = H, torso = T, legs = L, surface X left, surface Y left, surface Z left, surface X right, surface Y right, surface Z right)) including timely offset of the touch
  - By coding onset and offset the duration is also evaluated
  - Complexity (does the hand only touch one area or multiple areas in one touch)
  - Touches which are hidden (e.g. hand behind a leg) are coded as -999 as a touch may or may not happen in this time
  - Ambiguous Touches (due to 2D picture and camera angle it is not certain if the hand is touching or not) are coded as -777
  - Acoustic confounding factors (code duration of spoken word to account for confounding factors). Use an extra column for this.
  - Valid Coding time. Code the sequences which can be used for coding (no interaction, no distraction, correct posture, all limbs visible). Sequences of direct interaction with the child, use of pacifier etc. should not be coded, as it can affect the quantity of movement. If the infant shows a touch while using a pacifier or during parent interaction and the touch lasts longer than the distraction, the end of the touch marks the starting point to continue coding of the video (do not count the touch in which the distraction ends). Touches during which distractions happen should be left out from their onset until their offset, even if the distraction only occurs during parts of the touch.
- Complexity:
  - If the hand has moved from one area to another it is indicated with a dash (-). A dash can only be used if the hand was not lifted (e.g. T-L = hand was moved from torso to either one of the legs without losing contact to the body)
  - If one hand touches a border between two areas and it is unsure which area is being touched a slash (/) shall be used (e.g. T/H = hand is resting between torso and head)

## Rules

- A touch is counted as a touch, if it is more than a fingertip establishing contact with the body or surface (may be one finger, multiple fingers, whole hand, part of hand). The hand itself has to be involved in the touch, otherwise it is not counted as a touch (e.g. if the wrist touches the surface but the hand is lifted it does not count as a touch). Use shadows and wrist movement to helping identify a touch as well as its on- and offset.
- A touch starts (onset) as soon as the hand touches the body or surface. The touch ends (offset) as soon as contact between hand and body or surface is lost.
- A touch does not end if the hand is not lifted off the body or surface even if it moves.
- Each touch needs to have a minimum duration of 280ms (in our study 7 frames). If it is less than that, it does not count as a touch and should not be coded.
- Do not guess a touch. If the hand is blocked or cannot be seen use the code for an unknown touch (-999). This should also be done even if you can guess where the touch would be.

## Agreement between two coders

- Coding of both coders can be performed within the same Datavyu file using the function “hide columns” to being able to use the “temporal alignment” function
- If multiple coders analyse the videos and cross code the same video, there should be no more than 280ms difference between their on- and offsets (280ms error margin).
- If one coder A marks the location of a touch with “X” and the coder B with “X/Y” it is seen as an agreement. If coder A marks a touch as “X” and coder B as “X-Y” it is a disagreement (as multiple locations are touched). This rule also applies for sequences within a complex touch.
- If coder A codes a touch “X” with one single time code and coder B codes sequences “X” – “-999” – “X” it can be regarded as an agreement if:
  - on- and offsets between the sequences of coder B are no greater than 280ms
  - on- and offsets of coder A and those of the first and last sequence of coder B show no disagreement
  - location in all sequences of coder B agree with the location coded by coder A (except the unknown sequence)
  - the same rules apply for ambiguous sequences within a touch
- If coder A codes a whole touch as “-999” or “-777” while coder B marks it with a location, the touch should be removed from coding if coder A was the primary coder. If coder B was the primary coder, his/her code counts.
- If the primary coder did not code a touch because it was less than 280ms long and the other coder did code it, the touch should be removed from coding as the vote of the primary coder counts. If the situation is the other way around the touch should be included into analysis and usual agreement rules (max. 280ms difference for onset and offset) apply.
